# Investigation of invasive *Neisseria meningitidis* serogroup Y ST1466 case increases in New York State

**DOI:** 10.3389/fpubh.2025.1709761

**Published:** 2026-01-22

**Authors:** Krithivasan Sankaranarayanan, Andrew Peifer, Catharine Prussing, Elizabeth Owuor Bielli, Evan Owens, Kate Wahl, Anna Kidney, Wolfgang Haas, Aaditya Ojha, Caila B. Vaughn, Kimberlee A. Musser, Kara Mitchell

**Affiliations:** 1 Wadsworth Center, New York State Department of Health, Albany, NY, United States; 2 New York State Department of Health, Albany, NY, United States

**Keywords:** genomic epidemiology, invasive meningococcal disease (IMD), *Neisseria meningitidis*, pathogen genomics, public health surveillance, serogroup Y ST1466, whole genome sequencing (WGS)

## Abstract

Bacterial meningitis and septicemia caused by *Neisseria meningitidis* is a serious infection that requires immediate medical attention and prompt treatment. In the United States, cases of *N. meningitidis* serogroup Y increased sharply in 2023, leading to a CDC health advisory issued in March 2024, alerting public health agencies and healthcare providers of this surge. *N. meningitidis* serogroup Y is of particular concern because these strains demonstrate higher levels of resistance to the antimicrobials ciprofloxacin and penicillin. At the Wadsworth Center Bacteriology Laboratory (WCBL), all *N. meningitidis* isolates received are identified, serogroup determined by real-time PCR, and antimicrobial susceptibility testing is performed. A subset of isolates undergo whole genome sequencing (WGS) to determine the multilocus sequence type (MLST), presence of antimicrobial resistance genes, and relatedness analysis for outbreak investigations. In this study, we sequenced all 36 *N. meningitidis* serogroup Y isolates received between 2018 and 2024, of which 20 (~55%) were determined to be sequence type (ST) 1466. We used multiple bioinformatic methods to characterize the relatedness of isolates received at WCBL and compared them with ST1466 isolates from the United States with publicly accessible genomes. We identified a cluster of 8 cases of ST1466 *N. meningitidis* from the same region of New York State (NYS) that included a known transmission event linked to sharing a cigarette. Cases in this cluster were comprised of predominantly unvaccinated, African American, non-Hispanic, males between 45 and 76 years old, and mostly presented with sepsis; a demographic and clinical presentation not typical of those affected by *N. meningitidis* in the United States. Comparisons with publicly available genomes from ST1466 *N. meningitidis* strains from across the country showed that relatedness methods that mask genomic regions showing recombination signatures can obscure the diversity among these strains. This study, which importantly includes a case of known recent transmission, highlights the need to revisit genomic relatedness estimation and thresholds for defining relatedness in *N. meningitidis*. Our results also illuminate the importance of surveillance and characterization of *N. meningitidis* for future prevention and treatment, and the increased resolution that WGS provides to surveillance efforts.

## 1 Introduction

Invasive meningococcal disease (IMD) is a life-threatening illness caused by *Neisseria meningitidis*. Of the 12 serogroups of *N. meningitidis* identified, IMD in the United States (U.S.) is predominantly caused by serogroups B, C, and Y (1, 2). Although IMD may clinically manifest in a variety of ways, the most common and severe presentations are as meningitis and septicemia (3). In the U.S., IMD is fatal in 10 to 15% of cases, with case fatality impacted by *N. meningitidis* serogroup and sequence type (4, 5). Further, up to 20% of patients who survive IMD suffer from severe, long-term sequelae, including limb loss and neurological disorders (4, 6). Epidemiological surveillance of IMD is complicated by the transmission biology of *N. meningitidis*. An obligate human commensal, *N. meningitidis* is asymptomatically carried at rates that vary by age and setting (7, 8). While *N. meningitidis* is transmitted via respiratory droplets, it can accumulate mutations during nasopharyngeal carriage, resulting in genetic differences between the initially acquired strain and the isolate causing invasive disease (9–11).

Advances in whole-genome sequencing (WGS) and associated bioinformatics-analysis methods have made it an essential tool for epidemiological investigations (12–14). In addition to delineating transmission clusters, WGS enables assessment of virulence factors, antimicrobial resistance genes, and other clinically relevant genes, as well as exploring a pathogen’s evolutionary dynamics (15). However, genomic relatedness analysis in *N. meningitidis* is complicated by high rates of homologous recombination which increases genetic variability even among closely related strains (16). Further, while metrics have been proposed for identifying outbreak-associated *N. meningitidis* isolates, these vary depending on the specific bioinformatics approach utilized and are impacted by diversity within clonal complexes, recombination, and the heterogeneity of circulating strains (17, 18). These challenges are especially pronounced in community outbreaks involving multiple strains, requiring additional epidemiological evidence when interpreting the observed genetic distances (17).

The Wadsworth Center Bacteriology Laboratory (WCBL) at the New York State Department of Health (NYSDOH) receives and analyzes specimens from all suspected cases of bacterial meningitis in New York State (NYS). In addition to culture isolation and antimicrobial susceptibility testing, the WCBL routinely uses several laboratory-developed tests to determine the species and serogroup of common agents of bacterial meningitis (including *N. meningitidis*), and identify resistance genes by WGS (19–21). In 2023, a substantial increase in invasive *N. meningitidis* infections with similar demographic and risk factors was identified in the Central and Western Regions of NYS (CWNY) (New York State Regional Health Departments). Local health officials were advised of the regionwide increase and requested to report any common exposures to regional epidemiologists. Further investigations by county health officials identified two cases with symptom onset just 2 days apart in May 2023, with both individuals having lived in the same apartment building, acquainted with each other, and shared cigarettes during their exposure window. No additional common exposures were identified during these investigations.

Nationwide, similar increases in IMD were reported in 2023 and 2024 by the Centers for Disease Control and Prevention (CDC) (22), with serogroup Y sequence type (ST) 1466 accounting for a substantial proportion of cases. To improve surveillance and better understand the diversity of the *N. meningitidis* isolates associated with this surge in NYS, WGS was performed on all viable serogroup Y isolates obtained at WCBL during 2023 and 2024, along with a comparative group of isolates collected between 2018 and 2022. In this study, we describe the diversity, genetic relatedness, and epidemiological characteristics of the IMD-associated isolates from NYS and contextualize them within the broader national trend.

## 2 Materials and methods

### 2.1 Laboratory methods

As a reportable disease, specimens from all suspected cases of IMD in NYS are required to be sent to the WCBL for confirmatory testing. The WCBL determines the species and serogroups for all common causative agents of bacterial meningitis using real-time PCR assays developed in-house (23, 24). *Neisseria meningitidis* isolates were assigned to serogroups (B, C, Y, W, A) or reported as “non-serogroupable” (positive for *ctrA* and negative for serogroup-specific genes) or “non-typeable (non-capsulated)” (negative for *ctrA*). Susceptibility of *N. meningitidis* isolates to penicillin, ciprofloxacin, ceftriaxone, and azithromycin was determined by ETEST™. Susceptibility was interpreted according to the guidelines set by the Clinical Laboratory Standards Institute (25). Isolates were scored as susceptible, intermediate resistance, or resistant, for penicillin and ciprofloxacin, and as susceptible or non-susceptible for azithromycin and ceftriaxone. Isolates with a minimum inhibitory concentration (MIC) falling above the breakpoint for intermediate resistance but below the breakpoint for resistant were interpreted as being resistant. Whole-genome sequencing of *N. meningitidis* isolates was performed following our in-house protocol (26). Briefly, DNA was extracted from a total of 79 isolates (selected based on clinical/epidemiological need) using the QIAamp^®^ 96 DNA QIAcube^®^ HT kit. Illumina-compatible libraries were generated using the PCR-free Illumina^®^ DNA Prep kit (formerly Nextera Flex). Dual-barcoded libraries were pooled and sequenced on Illumina^®^ NextSeq500/NextSeq550 instruments.

### 2.2 Bioinformatics methods

Raw sequencing reads were quality-filtered using Trimmomatic (v0.39) (27) followed by *de novo* assembly using the SPAdes assembler (v3.15.5) (28). The resulting genome assemblies were processed using custom scripts to retain high-quality contigs (length > = 500, coverage > = 5x). Assembly quality was evaluated using BUSCO (v5.8.2) (29). In-silico multilocus sequence typing was performed using ‘mlst’ (v2.23.0, 2024-10-01 database) (30, 31). Gene prediction and annotation was performed using Bakta (v1.11.3) (32). Prediction of virulence factors and antimicrobial resistance genes was performed using our in-house clinical pipeline (19). Additionally, allelic variants for antimicrobial resistance genes were determined using BLASTN searches (33) against the corresponding gene-specific sequence databases obtained from PubMLST (30), with allele assignment requiring 100% query coverage, 100% target coverage, and 100% nucleotide identity.

In-silico serogroup prediction was performed using an in-house tool developed based on the pmga pipeline (34, 35). A custom reference database comprising allele sequences for 40 genes associated with capsule biosynthesis (Region A), transport (Region C), and translocation (Region B) across serogroups A, B, C, Y, W, and X was generated from PubMLST. For each isolate, predicted gene sequences were compared against this database using BLASTN to identify capsule polysaccharide synthesis genes and serogroup-specific markers (35). Serogroup assignment required recovery of full-length genes across all three regions. Where applicable, allele variants for the *ctrA* gene and serogroup-specific marker genes were determined using the same criteria described for the antimicrobial resistance genes. The results of the genome analyses are summarized in Supplementary Table S1.

Quality-filtered reads and genome assemblies were used as input for our in-house relatedness analysis pipeline (36). Briefly, genomes were grouped into clusters using Mash (v2.1) (37), followed by cluster refinement using pairwise distances calculated from a core-genome tree (Parsnp, v1.5.6) (38). Within each cluster, single nucleotide polymorphisms and short insertions/deletions, collectively referred to as ‘mutation events’, were identified for each isolate in comparison to a single genome representative for that cluster (Samtools v1.9, Bcftools v1.17, FreeBayes v1.3.6) (39, 40). Pairwise mutation-event matrices were generated to evaluate genomic relatedness between isolates within a cluster. Relatedness analysis was also performed using previously published approaches (17). Briefly, isolate genomes were grouped into clusters using PopPUNK (v2.7.5, Neisseria_meningitidis_v1_refs database) (30, 41). Within each cluster, pairwise SNP distances between isolates were generated using reference-alignment based (SKA2 v0.4.0, Gubbins v3.3.5) (42, 43) and reference-independent methods (kSNP3 v3.1) (44).

### 2.3 Epidemiological methods

Invasive infection with *Neisseria meningitidis* is reported to local health departments in NYS through the Electronic Clinical Laboratory Reporting System. Upon report, each case is investigated to collect demographic, clinical, and exposure information and to assess for contacts in need of post-exposure prophylaxis. Case investigations are conducted via telephone using a standardized, disease-specific questionnaire and recorded in New York State’s and New York City’s area-specific Communicable Disease Electronic Surveillance System. NYSDOH epidemiologists across four regional health departments in NYS and New York City monitor these investigations and provide guidance to county (geographical and administrative subdivision of the state) health officials as appropriate.

## 3 Results

### 3.1 Characteristics of *N. meningitidis* isolates received at WCBL

**Figure 1:**
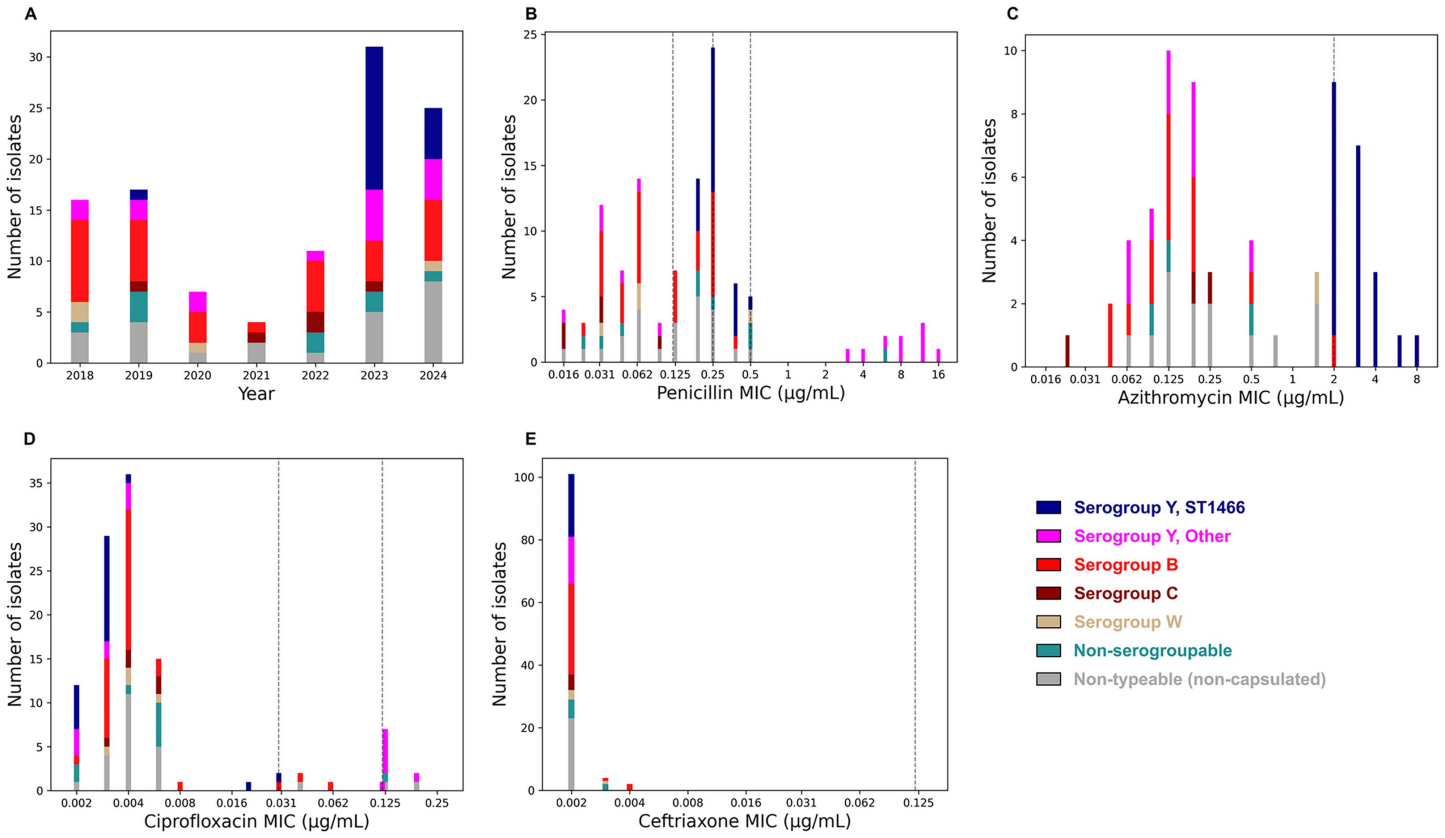
Characterization of *N. meningitidis* isolates received at WCBL from 2018–2024. Isolates groups are color-coded to reflect serogroup assignments. Serogroup Y ST1466 isolates are placed in a distinct category from other serogroup Y isolates. **(A)** Total yearly isolate counts stratified by serogroup. **(B–E)** Antimicrobial susceptibility testing results for isolates against four antibiotics: **(B)** penicillin (*n* = 109) (21), **(C)** azithromycin (*n* = 63), **(D)** ciprofloxacin (*n* = 109), and **(E)** ceftriaxone (*n* = 107). For each antibiotic, CLSI breakpoints for susceptible, intermediate resistance, and resistance are depicted (25).

Between January 2018 and December 2024, the WCBL received 139 bacterial isolates for *N. meningitidis* testing from hospital and public health labs in NYS. Of these, 111 unique isolates were confirmed as *N. meningitidis* and assigned serogroups by real-time PCR. Two isolates were nonviable and could not undergo further testing. Overall, 36 *N. meningitidis* isolates were identified as belonging to serogroup Y (Supplementary Table S1). Additionally, following the decline in cases during 2020–2021 due to COVID-19 quarantine measures, a sharp increase in serogroup Y isolates was observed in 2023 and 2024, with 28 of the 36 isolates received in this period (Figure 1A) (45, 46).

**Figure 2:**
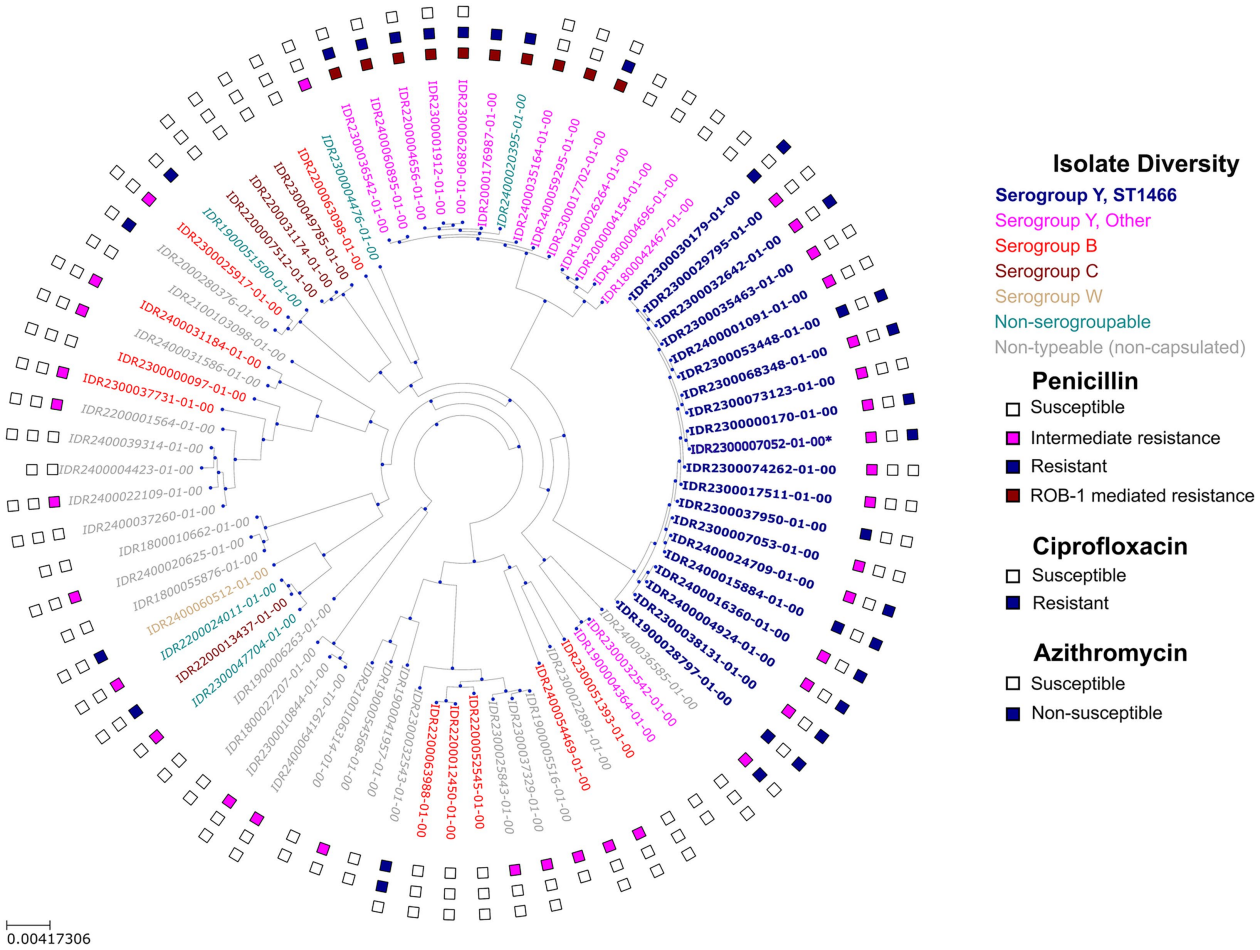
Maximum likelihood phylogenetic tree depicting diversity of *N. meningitidis* isolates sequenced at WCBL. Isolate labels are color coded to reflect serogroup assignments. Serogroup Y ST1466 isolates are placed in a distinct category from other serogroup Y isolates. Boxes adjacent to isolate labels denote resistance interpretation from ETEST. Innermost box: penicillin, middle box: ciprofloxacin, and outermost box: azithromycin. *Isolate IDR2300007052-01-00 is assigned to ST17554 and differs from the ST1466 isolates by one allele.

Genome sequencing and phylogenetic analysis were performed on 79 isolates comprising all serogroup Y isolates, and a subset of serogroup B, serogroup C, and non-serogroupable/non-typeable (non-capsulated) isolates. MLST analysis identified 30 STs among 77 unique isolates (two isolates were not assigned to any ST). Serogroup Y ST1466 (*n* = 20; including one closely related isolate, ST17554) and serogroup Y ST3587 (*n* = 10; including one isolate lacking serogroup markers) collectively accounted for the largest fraction (~38%) of isolates sequenced (Figure 2).

Of the 109 *N. meningitidis* isolates tested for susceptibility to penicillin, 45 showed intermediate resistance (MIC 0.125 to 0.25 μg/mL) and 21 isolates were resistant (MIC 0.38 to 16 μg/mL) (Figure 1B) (21). Specifically, all ST1466 isolates showed reduced susceptibility to penicillin (MIC 0.19 to 0.5 μg/mL) and carried a mosaic *penA* gene (PubMLST locus NEIS1753, alleles 335 and 3644) associated with this phenotype (Supplementary Table S1) (47). Similarly, all ten ST3587 isolates contained the *bla*_ROB-1_ gene and were the most resistant to penicillin (MIC 3 to 16 μg/mL) (Figure 2) (48). All *N. meningitidis* isolates tested (*n* = 107) were susceptible to ceftriaxone (MIC ≤ 0.004 μg/mL) (Figure 1E).

Ciprofloxacin resistance (MIC 0.125 to 0.19 μg/mL) was observed in 10 *N. meningitidis* isolates (total tested = 109), of which eight belonged to ST3587 (Figure 2). All resistant isolates had mutations in the *gyrA* gene (T91I n = 10, T173A n = 7; PubMLST locus NEIS1320, alleles 1717, 3309, and 908) associated with fluoroquinolone resistance (49) (Supplementary Table S1). All ST1466 isolates were susceptible to ciprofloxacin (MIC ≤ 0.03 μg/mL) (Figure 1D). Resistance to azithromycin was primarily observed among ST1466 isolates with 12 non-susceptible isolates (MIC 3 to 8 μg/mL) and 8 isolates with reduced susceptibility (MIC 2 μg/mL) (Figure 1C). Isolates with azithromycin MIC ≥ 0.75 μg/mL contained the K823E mutation in the *mtrD* gene (PubMLST locus NEIS1633, alleles 1730, 4108, 5016, 5029, and 5764), which has been correlated with reduced susceptibility to this antibiotic in *Neisseria* species (Supplementary Table S1) (50, 51).

### 3.2 Genomic diversity of *N. meningitidis* serogroup Y ST1466 isolates

**Figure 3:**
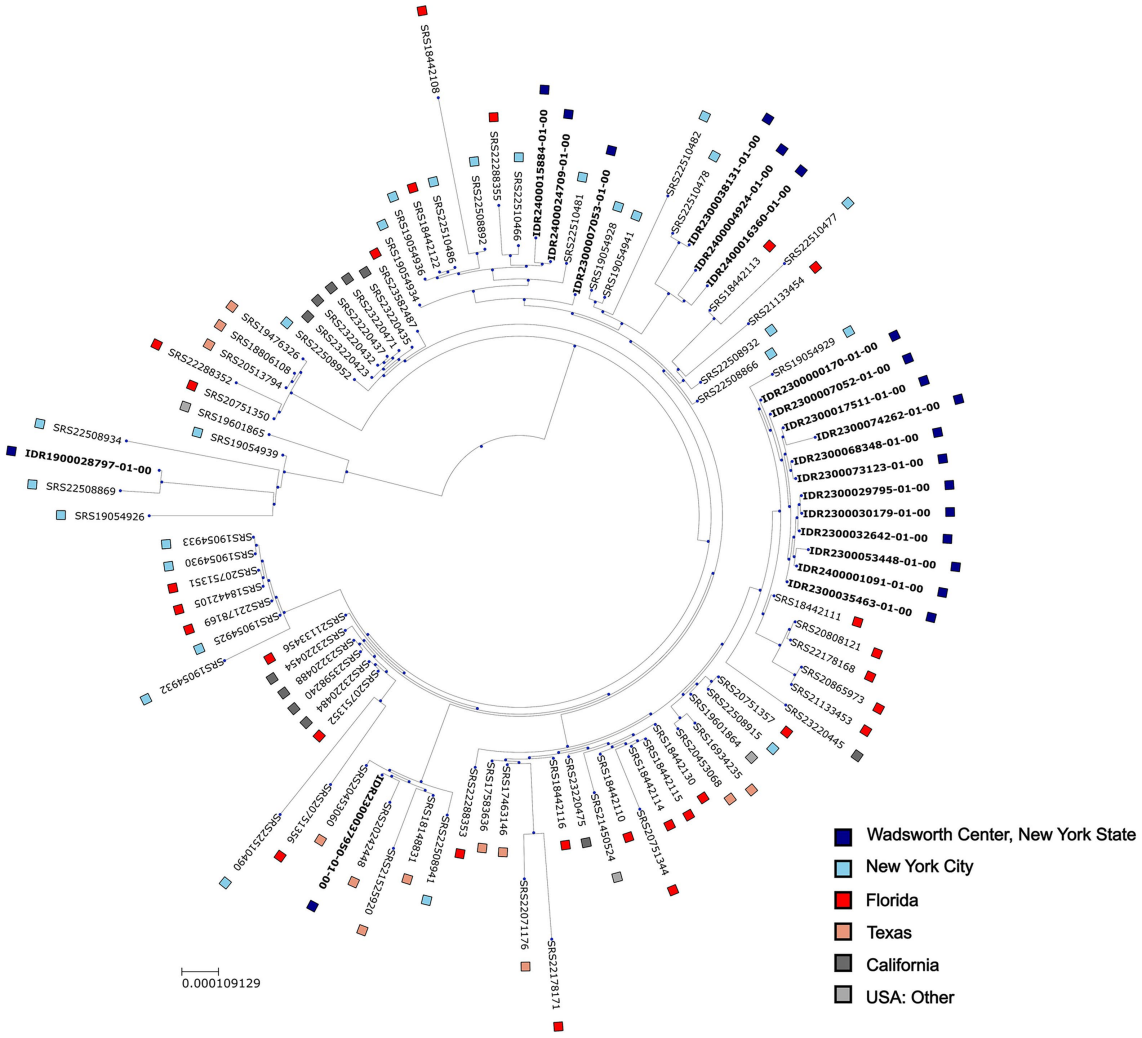
Maximum likelihood phylogenetic tree depicting diversity of *N. meningitidis* serogroup Y ST1466 isolates from WCBL. For comparison, ST1466 isolates from the NCBI Pathogen Detection database are also included. Isolates from NCBI were retrieved from the following BioProject accessions: PRJNA1020819 (New York City), PRJNA1142047 (California), PRJNA1170207 (Texas), PRJNA934874 (Florida), and PRJNA991232.

All 79 isolates sequenced at WCBL and a collection of 285 *N. meningitidis* isolate genomes submitted by public health laboratories in the U.S. between January 2023 and December 2024 (corresponding to the nationwide increase in serogroup Y, ST1466) were processed using a relatedness-analysis pipeline developed at WCBL (36). A total of 302 isolates (WCBL *n* = 60, NCBI *n* = 242) were grouped into 32 genomic clusters while the remaining isolates were singletons. Genomic clusters showed concordance with MLST assignments. All 100 ST1466 isolates (WCBL *n* = 20, NCBI *n* = 80) were placed into a single genomic cluster. Phylogenetic analysis of the ST1466 isolates identified 12 WCBL isolates from the CWNY region that formed a distinct clade (Figure 3).

**Figure 4:**
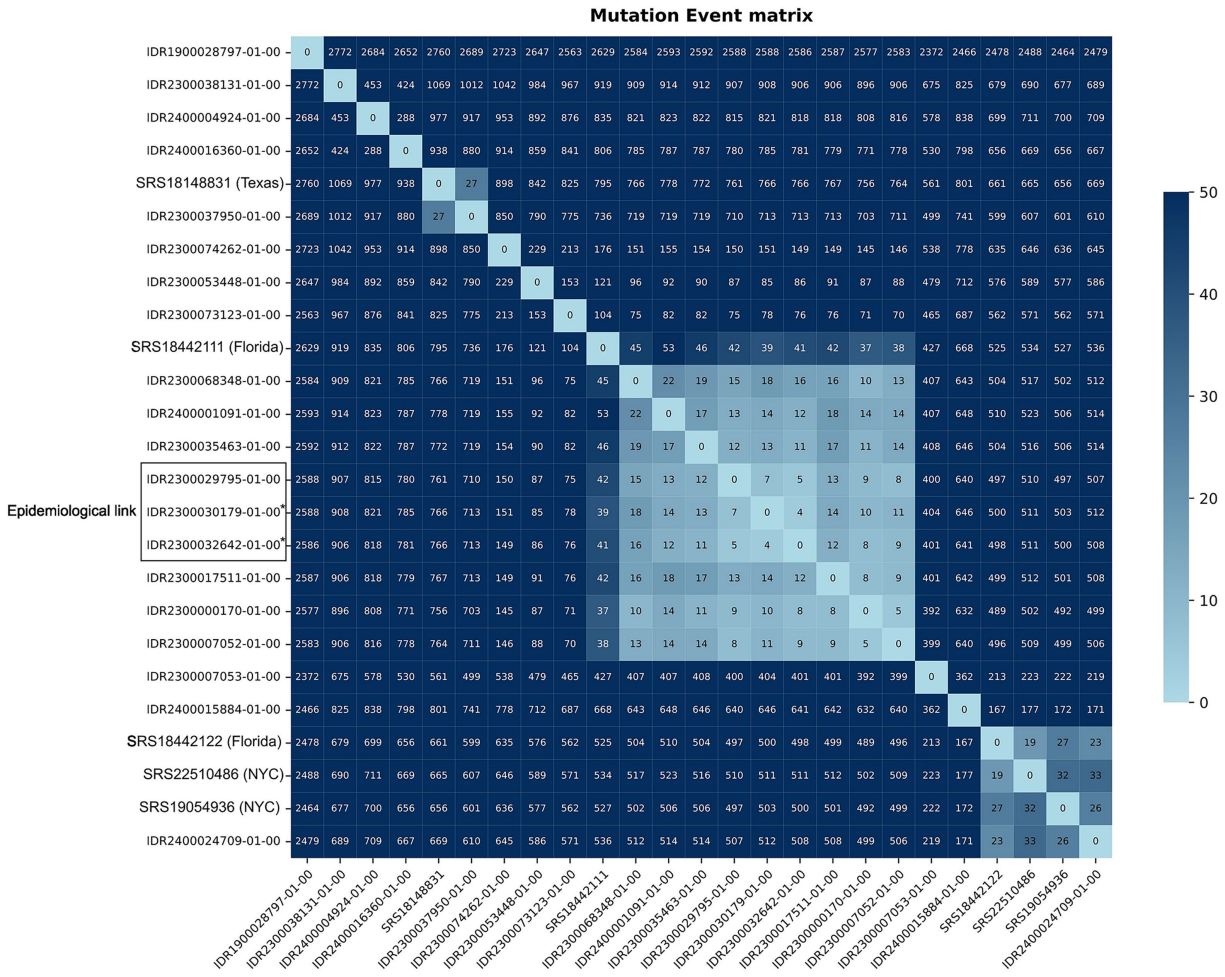
Mutation event matrix depicting pairwise genetic distances among *N. meningitidis* ST1466 isolates from WCBL. For comparison, ST1466 isolates from the NCBI pathogen detection database that are separated by fewer than 50 mutation events from a WCBL isolate are also included. *Two isolates submitted from the same individual.

**Figure 5:**
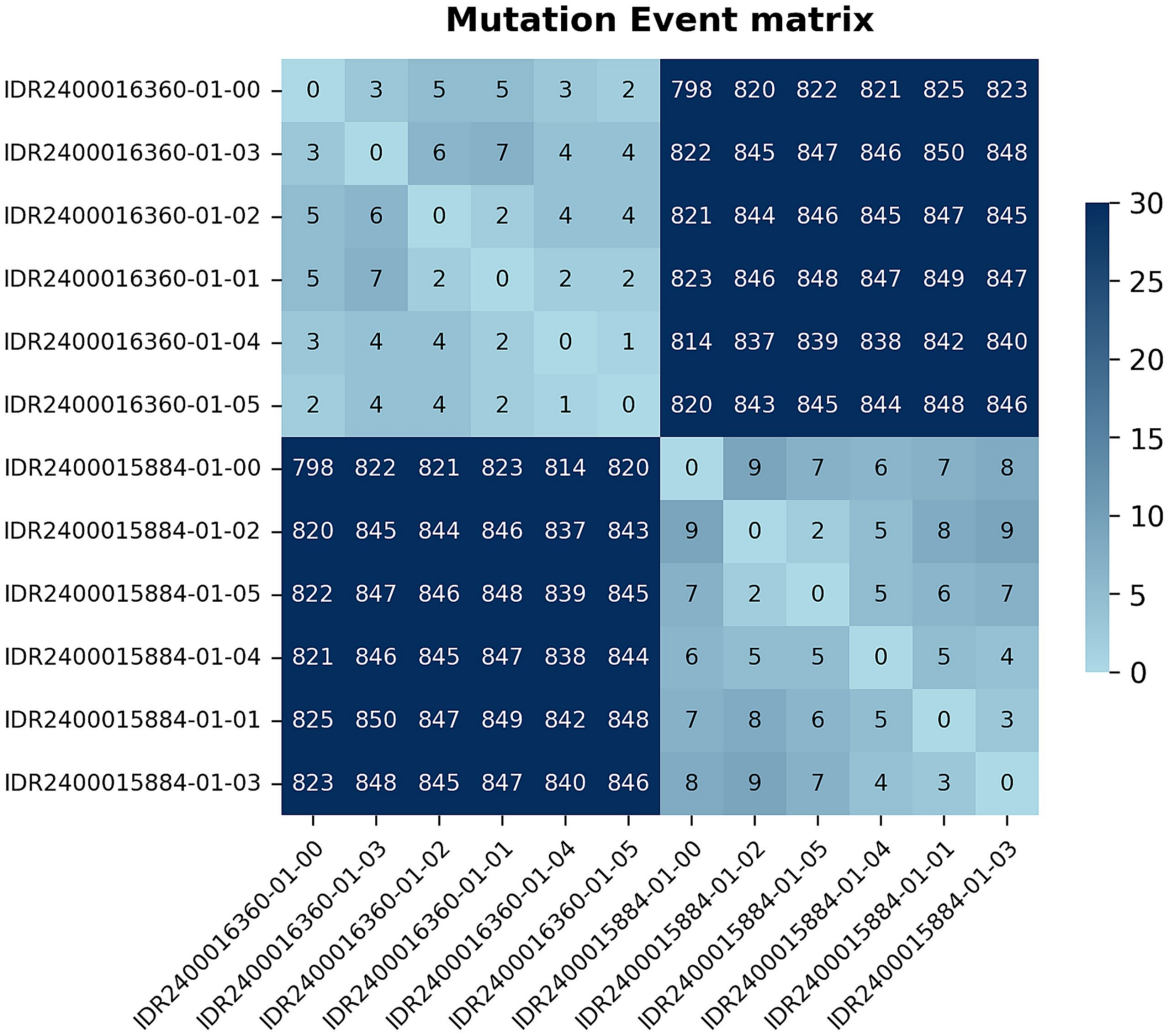
Mutation event matrix depicting within-specimen pairwise genetic distances for two *N. meningitidis* ST1466 isolates from WCBL. For each specimen, data is included for isolate genomes obtained from five independent single-colony picks (labels ending in −01 through −05). Sequence data for the original isolate specimen is also included (label ending in −00).

Of these, a subset of nine CWNY isolates (eight cases) were found to be closely related to each other based on genome-wide pairwise differences (4–22 mutation events) (Figure 4). This group includes three isolates obtained from the two individuals with a known transmission link, with the isolates differing by 4–7 mutation events from each other and by 8–18 mutation events from the remaining six isolates in this group (Table 1). Further, the CWNY isolates in this group differ by 38–53 mutation events from the nearest non-WCBL ST1466 isolate (obtained from NCBI) (Figure 4). To contextualize the pairwise distances observed for the epidemiologically linked isolates and to characterize within-specimen variability, we grew *N. meningitidis* cultures from multiple single-colony picks for two ST1466 specimens. Pairwise differences of between 1 and 9 mutation events were observed between isolates cultured from the same specimen (Figure 5), overlapping with the mutation event range observed for the epidemiologically linked isolates (Table 1).

**Table 1:** Within and between group variation in genomic distance as determined by four relatedness-analysis pipelines.

Isolate group	Comparative group	[1]	[2]	[3]	[4]
Epidemiologically linked isolates (*n* = 3)	Within-group	4–7	9–13	4–5	3–4
	(Other) CWNY group (*n* = 6)	8–18	12–22	7–14	7–14
	(Other) NYS ST1466 (*n* = 11)	75–2,588	15–79	18–536	11–70
	NCBI ST1466 (*n* = 80)	39–2,986	17–107	21–586	10–76
CWNY group (*n* = 9)	Within-group	4–22	9–25	4–21	3–20
	(Other) NYS ST1466 (*n* = 11)	70–2,597	14–79	13–538	9–73
	NCBI ST1466 (*n* = 80)	37–2,993	15–115	21–589	10–81
IDR2400015884	Single-colony picks (*n* = 5)	2–9	8–16	3–9	3–9
IDR2400016360	Single-colony picks (*n* = 5)	1–7	7–16	3–7	3–7

[1] WCBL in-house pipeline, [2] reference-independent pipeline (kSNP3; k-mer length 251), [3] reference-based pipeline without recombination masking (SKA2), and [4] reference-based pipeline with recombination masking (SKA2 + Gubbins). The WCBL pipeline reports mutation events (SNPs and short insertion and deletions) while the remaining three approaches report SNP differences. Sequencing data for the NCBI isolates were obtained from BioProject accessions: PRJNA1020819 (New York City), PRJNA1142047 (California), PRJNA1170207 (Texas), PRJNA934874 (Florida), PRJNA991232. Data was obtained from isolates submitted during 2023 and 2024.

As a comparison, our 302 *N. meningitidis* isolate genomes were analyzed using relatedness methods described in prior CDC studies of *N. meningitidis* outbreaks (17, 18). Consistent with the WCBL pipeline, all 100 ST1466 isolates were placed into a single genomic cluster by PopPUNK (41). Pairwise distances for epidemiologically linked isolates were between 4 and 5 SNPs for reference-based comparisons and 3–4 SNPs when recombination filtering is applied. Similarly, for the CWNY group, pairwise distances ranged from 4 to 21 SNPs (reference-based) and 3–20 SNPs (recombination-filtered), respectively. Compared to the reference-based approach, the reference-independent pipeline (kSNP3 with k = 251) yielded higher distances for both the epidemiologically linked (9–13 SNPs) and CWNY groups (9–25 SNPs) (Table 1). Similar trends were observed when analyzing isolates collected to characterize within-specimen variation.

The eight CWNY serogroup Y cases in this group ranged in age from 45 to 76 years (median age 57 years). Seven individuals were non-Hispanic Black (male = 6, female = 1) and the remaining individual was a non-Hispanic White female. Illicit substance use was reported by five of the cases, and all but one were current smokers. For the two cases with a known transmission link, symptom onset was within one day of each other. None of the cases presented with meningitis; five of the cases presented with bacteremia alone, while others presented with pneumonia, septic shock, septic arthritis and epiglottitis. None of the cases had documentation of receiving a serogroup Y containing meningococcal vaccine. Of these eight cases, seven survived. Epidemiological characteristics of the NYS ST1466 cases are summarized in Table 2.

**Table 2:** Epidemiological summary of NYS ST1466 cases.

Characteristics	CWNY	Other NYS
Cases	8	11
Region		
Capital district	0	2
Central	2	0
Western	6	4
Metropolitan Area	0	5
Race		
Black or African American, Non-Hispanic	7	3
White, Hispanic	0	1
White, Non-Hispanic	1	4
White, Unknown	0	1
Unknown	0	2
Sex		
Male	6	9
Female	2	2
Age		
Average	58	55
Median	57	49
23–26 years	0	1
27–64 years	5	7
>65 years	3	3
Smoker		
Current	6	3
Marijuana	1	0
None	1	6
Unknown	0	2
Substance use		
Yes	5	3
No	3	6
Unknown	0	2
Outcome		
Alive	7	8
Deceased	1	1
Unknown	0	2
Source		
Blood	8	9
Body fluid	0	1
Urine	0	1

## 4 Discussion

Consistent with national trends, an increase in IMD cases caused by serogroup Y was observed in NYS between 2023 and 2024 (22), with most isolates identified as ST1466 (*n* = 18) or ST3587 (*n* = 7). Among serogroup Y-positive specimens, approximately 85% were cultured from blood and obtained from patients ranging in age from 24 to 81 years (median age 48.5 years) (Supplementary Figure S1). The serogroup Y isolates were primarily received from counties in metropolitan NY and CWNY, with ST1466 isolates predominantly obtained from CWNY (Supplementary Figure S2). The distribution of ST1466 cases based on age (median age 56 years), race (58% non-Hispanic Black persons), smoking status (58% smokers), and vaccination status (all 8 cases unvaccinated) is similar to patterns reported in other jurisdictions, such as Virginia (median age 47 years, 77% non-Hispanic Black persons, 63% smokers, and 35 out of 36 cases unvaccinated) (52), and nationwide by the CDC (22). Further, ST1466 cases had atypical clinical presentation in NYS (bacteremia and bloodstream infections, septic arthritis, and pneumonia) and Virginia (bloodstream infections, and nonspecific symptoms including fever, nausea, muscle aches), with meningitis being rarely observed (52). Overall, these similarities in epidemiological and clinical trends indicate patterns that may more broadly apply to serogroup Y IMD in the United States, rather than to characteristics specific to ST1466.

Our relatedness analysis, coupled with epidemiological data, identified eight unique ST1466 cases (nine isolates) that were closely related (CWNY group, 4–22 mutation events) (Table 1), including two cases (three isolates) with a known transmission link. Further, the CWNY group is clearly distinguished from the other NYS ST1466 isolates (>70 mutation events), and non-WCBL ST1466 isolates (>39 mutation events). While pairwise distances for the CWNY group are consistent across the multiple bioinformatic methods, the recombination-filtering approach results in marked decreases in distances for divergent isolate pairs in the ST1466 cluster (Figure 3; Table 1). This is explained by the total number of variant sites used for pairwise comparisons (WCBL pipeline: ~17,000 sites, reference-based with recombination filtering: ~820 sites). The significant decrease in variant sites following recombination filtering can obscure genetic differences, which may contribute to spurious associations between distantly related isolates (53).

In conclusion, our WGS analysis showed that the increase in NYS IMD cases was driven by serogroup Y ST1466 *N. meningitidis*, consistent with the national trends. In addition to identifying a closely related group of eight cases from CWNY, our relatedness analysis showed that the NYS ST1466 isolates are genetically distinct from isolates submitted by other U.S. public health laboratories during the 2023–2024 nationwide surge. The NYS ST1466 isolates were intermediately resistant or resistant to penicillin and exhibited reduced susceptibility to azithromycin. Azithromycin is one of the antimicrobials recommended for prophylaxis for close contacts of IMD patients in regions where ≥20% of IMD samples show resistance to ciprofloxacin (54). To our knowledge, this is the first report of an outbreak of azithromycin non-susceptible *N. meningitidis* in the United States. Together, these findings underscore the importance of integrating WGS-based surveillance with robust epidemiological data to guide meningococcal disease detection, prevention, and treatment.

## Data Availability

The datasets presented in this study can be found in online repositories. The names of the repository/repositories and accession number(s) can be found at: https://www.ncbi.nlm.nih.gov/, PRJNA1032916.
