# Supplementary material for: Investigation of invasive Neisseria meningitidis serogroup Y ST1466 case increases in New York State
Source: Front Public Health. 2026 Jan 22;13:1709761. doi: 10.3389/fpubh.2025.1709761 (PMC12872775; doi:10.3389/fpubh.2025.1709761)
Supplement: Supplementary file 1 [file Supplementary_file_1.docx]

Supplementary Material

# Supplementary Figures


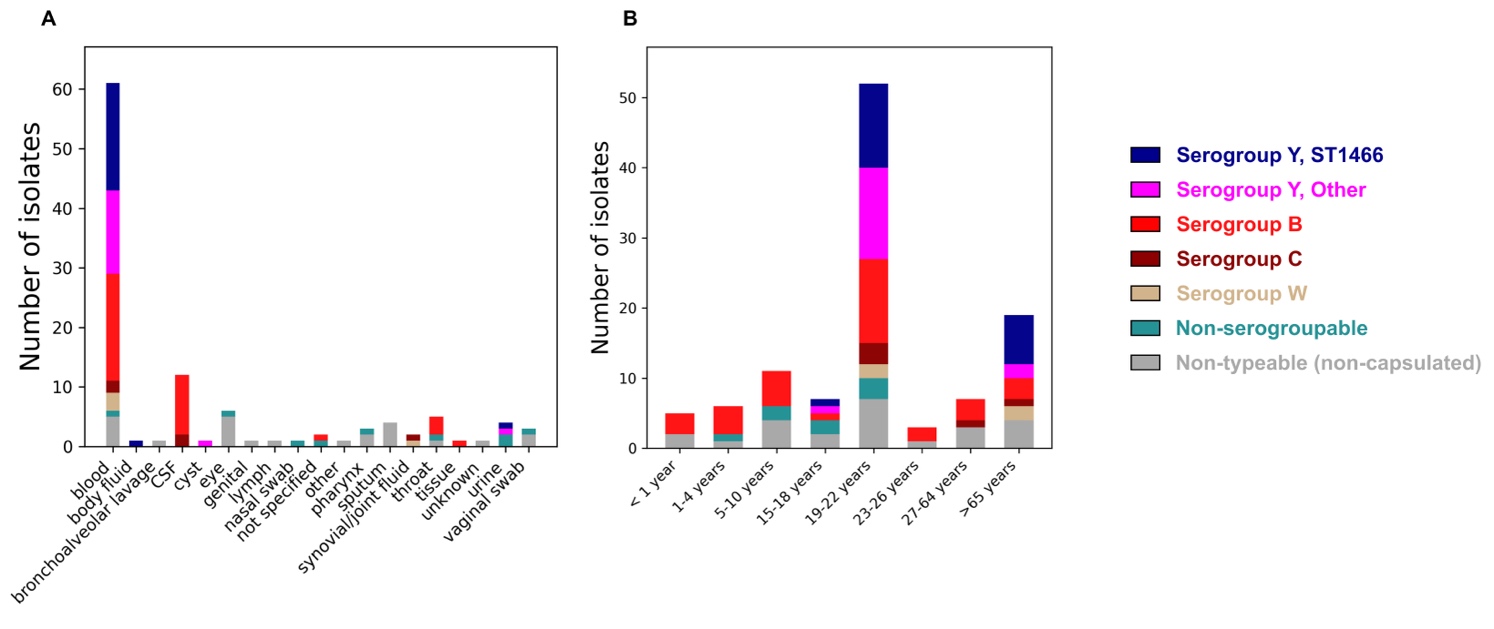


**Supplementary Figure 1.** Distribution of WCBL *N. meningitidis* isolates based on (A) collection source, and (B) age. Isolates are color-coded to reflect serogroup assignments. Serogroup Y ST1466 isolates are depicted as a separate category from other serogroup Y isolates.

**
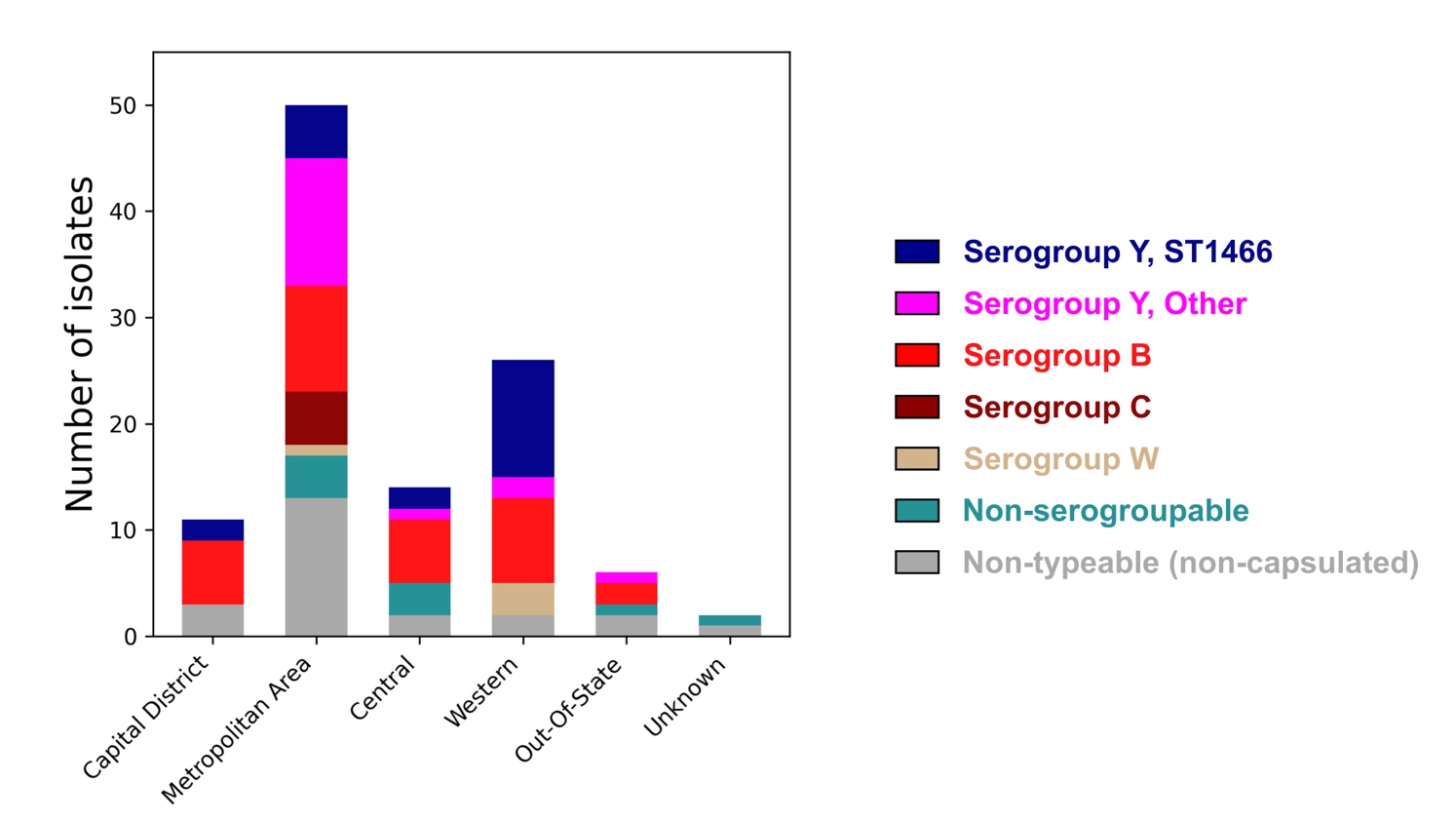
**

**Supplementary Figure 2.** Distribution of WCBL *N. meningitidis* isolates based on collection region. Serogroup Y ST1466 isolates are depicted as a separate category from other serogroup Y isolates.
